# RNA-binding protein CELF1 promotes tumor growth and alters gene expression in oral squamous cell carcinoma

**DOI:** 10.18632/oncotarget.6204

**Published:** 2015-10-20

**Authors:** Reniqua P. House, Sudha Talwar, E. Starr Hazard, Elizabeth G. Hill, Viswanathan Palanisamy

**Affiliations:** ^1^ Department of Oral Health Sciences and Center for Oral Health Research, College of Dental Medicine, Medical University of South Carolina, Charleston, SC, USA; ^2^ Division of Bioinformatics, Medical University of South Carolina, Charleston, SC, USA; ^3^ Department of Public Health Sciences, Medical University of South Carolina, Charleston, SC, USA

**Keywords:** oral squamous cell carcinoma, CELF1, mRNA splicing and mRNA turnover

## Abstract

The RNA binding protein CELF1 (also known as CUGBP1) is emerging as a critical regulator of cancer cell proliferation and apoptosis. Here, to provide a global prospective of CELF1 regulation of oral squamous cell carcinoma, we performed RNA-sequencing in oral cancer cells and CELF1 overexpression analysis in non-malignant human oral keratinocytes. Our approaches identified 1283 mRNAs differentially regulated as a function of CELF1 expression and more importantly CELF1 promoted alternative splicing of several target pre-mRNAs, which are known to be involved in various cancer biological processes. Overexpression of CELF1 in non-malignant human oral keratinocytes protected cells against oxidative damage and altered gene expression patterns. Finally, we provide evidence that reduction of CELF1 protein using a xenograft tumorigenesis mouse model decreased tumor growth. Altogether, these data provided a comprehensive view of the CELF1 mRNA regulatory network in oral cancer and suggests that CELF1 and/or its target mRNAs are viable candidates for therapeutic intervention.

## INTRODUCTION

The human genome consist of approximately 424 predicted RNA binding proteins (RBPs), and only a few have been extensively characterized for their role in cancer [1]. RBPs are critical regulators of co- and post- transcriptional gene expression and are capable of associating with both messenger RNAs and non-coding RNAs [2]. RBPs associate with their mRNA targets by binding to specific sequence motifs and/or recognizing distinct RNA secondary structures [3]. As a result, RBPs play major roles in mRNA metabolism including splicing, polyadenylation, capping, export, localization, translation and turnover [4, 5]. The RNA-binding activity and the expression level of RBPs can be rapidly modulated in response to external stimuli, via post-translational modifications [6]. Consequentially, deregulation of RBPs can lead to cancer progression. For example, the RBP HuR is implicated in tumorigenesis and tumor cell survival [7, 8]. SRSF1, a splicing factor, is phosphorylated in cancer and has been implicated in cellular transformation [9]. Lastly, AUF1 regulates epithelial-mesenchymal transition and modified AUF1 activity, promotes cancer progression in various tissues [10, 11]. Interestingly, altered expression of RBPs are noted in oral squamous cell carcinoma (OSCC) [8, 12, 13], raising the possibility that disruption of post-transcriptional regulation may contribute to oral cancer tumorigenesis.

CUGBP ­embryonic lethal abnormal vision-like family member 1 (CELF1) otherwise called CUGBP1, is a ∼50kDa member of the ELAV-like family of RNA binding proteins. Both biochemical and cell-based studies indicate that CELF1 preferentially binds to GU-rich elements (GREs) predominantly located in the 5′ and 3′ UTRs (untranslated regions) of mRNAs [14-17]. Bioinformatic analysis of the human transcriptome revealed that at least 5% of human transcripts contain GRE motifs and these mRNAs are involved in cellular functions such as: nucleic acid metabolism, protein modification and cell proliferation [18]. When CELF1 associates with its mRNA targets, it can influence their alternative splicing, translation and turnover [19-21]. CELF1 is primarily studied for its contributory role in myotonic dystrophy type 1 (DM1) disease progression [22-25] however, recent emerging evidences support CELF1 as a potential regulator of cancer progression [26-28]. In HeLa cells, Ribonucleoprotein Immuno-Precipitation-microarray (RIP-Chip) studies revealed that CELF1 associates with GRE containing mRNAs, that encoded proteins involved in apoptosis, cell proliferation and cell motility [17]. In Non-Small Cell Lung Cancer (NSCLC), CELF1 protein expression correlates with poor patient survival [26, 29]. In addition, reduction of CELF1 using siRNA in lung cancer cells decreased the proliferative rate and the capacity of the lung cancer cells to form colonies [29, 30]. Lastly, we have observed in head and neck cancer, that CELF1 protein is over expressed in human squamous cell carcinoma cell lines and tissue specimens in comparison to normal epithelium [27]. Moreover, reduction of CELF1 in oral cancer cells decreased cell growth and increased apoptosis, suggesting that CELF1 may be an important regulator of oral cancer progression [27].

High throughput sequencing cross-linked immunoprecipitation (HITS-CLIP) and RIP-Chip CELF1 studies have identified CELF1 nucleotide recognition sequences and CELF1 associated mRNA targets [14, 17, 31, 32]; however, the CELF1 positively and negatively controlled mRNAs and the CELF1-mediated alternative splicing events in cancer remains to be determined. Therefore, we set out to identify the CELF1 regulatory network in oral cancer cells. Using next generation sequencing (RNA-seq) we identified 1283 CELF1 regulated mRNAs in oral cancer cells associated with cell proliferation, angiogenesis and signal transduction. In addition, we determined that CELF1 promoted the alternative splicing of 282 pre-mRNAs. In an inducible shRNA xenograft mouse model, we demonstrated that the loss of CELF1 expression resulted in reduced tumor burden. Finally, overexpression of CELF1 in immortalized human oral keratinocytes enhanced cell survival to oxidative damage and augmented EGFR signaling. Altogether, these data support CELF1 as a major contributor to oral squamous cell carcinoma tumorigenesis.

## RESULTS

### CELF1 influences the expression of hundreds of mRNAs encoding proteins involved in tumor growth and malignancy

**Figure 1:**
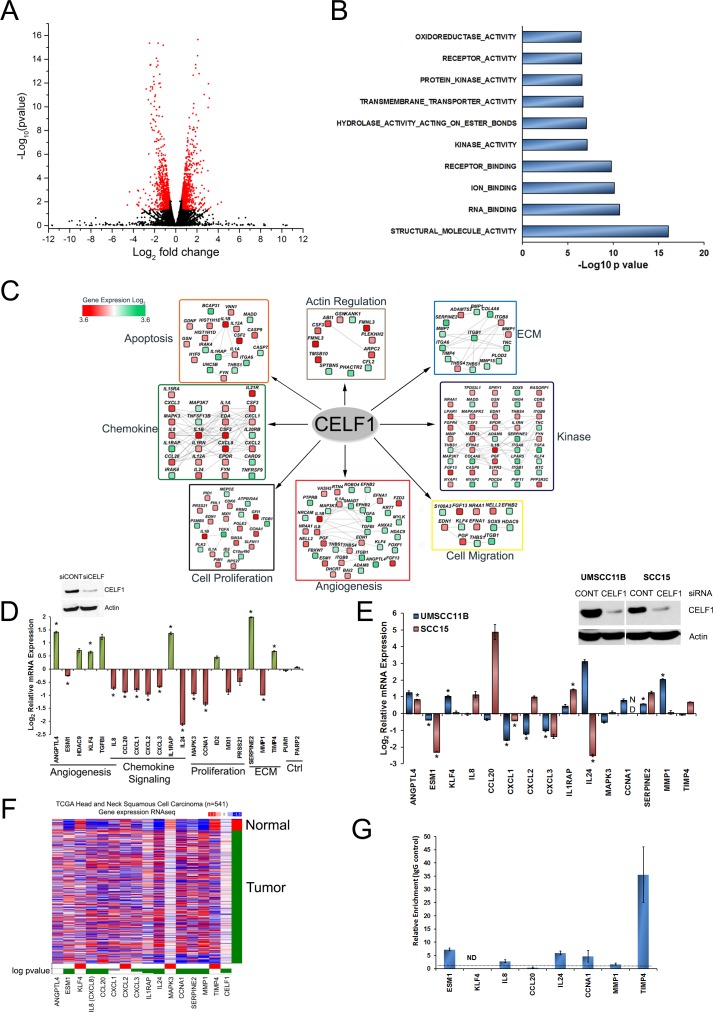
Next generation sequencing (RNA-seq) identifies novel targets regulated by CELF1 **A.** Volcano plot of the 1283 significant differentially regulated mRNA transcripts (shown in red). **B.** GO (gene ontology) significantly enriched molecular function analysis of CELF1 controlled mRNAs. **C**. Biological process enrichment analysis of up (red) and down (green) CELF1 regulated transcripts. **D.** Validation of RNA-seq mRNA targets using qRT-PCR as a function of CELF1 expression. Down regulated transcripts (red); up regulated transcripts (green); neutral transcripts (gray). Bars represent mean ± SE; *N* = 3. *p value < 0.05. **E.** Validation of RNA-seq mRNA targets using qRT-PCR as a function of CELF1 expression in UMSCC-11B and SCC15 OSCC cell lines. Bars represent mean ± SE; *N* = 3. **p value < 0.05. **F.** Analysis of mRNA levels for the 15 validated mRNA targets using UCSC cancer genomics browser. TCGA HNSCC datasets were normalized and represented as a heatmap. Red: up regulated; blue: down regulated. Targets significantly upregulated in normal tissues (red); significantly upregulated in tumor tissues (green). Wilcoxon statistical analysis. **G.** Ribonucleoprotein immunoprecipitation (RNP-IP) of CELF1 associated mRNAs quantified using qRT-PCR. ND: Not detected in CELF1 immunolysates.

As CELF1 is known to control post-transcriptional gene expression [19-21], we expect that deletion of CELF1 would perturb gene expression, through either positive or negative regulation of target mRNA expression. To elucidate the post-transcriptional regulation of CELF1 in oral cancer progression, we sequenced total RNA isolated from UMSCC-74B cells 48 hrs post transfection with control or siRNA targeting CELF1. Differential gene expression analysis, determined that the relative levels of 1283 mRNA transcripts corresponding to 1174 genes were significantly affected by CELF1 protein expression (Figure 1A and Table S1). As our analyses have identified both direct and indirect mRNA targets of CELF1, next, we analyzed the 3′UTRs of our 1174 genes for the presence of GREs. We searched the 3′UTRs for the heptamer sequence TGTXTGT (X = any nucleotide) shown to specifically interact with high affinity to CELF1 *in vitro* [33] and the top 20 hexamer sequences enriched in the 3′UTRs of CELF1 controlled mRNAs identified in C2C12 cells [14]. Overall, we have observed 86% (1009/1174) of genes contain at least 1 hexamer sequence and 41% (481/1174) of genes contain at least 1 heptamer sequence in their 3′UTR (Table S2). Gene ontology (GO) molecular function enrichment analysis using the cytoscape plugins Cluepedia and ClueGO, revealed that the CELF1 regulated mRNAs in oral cancer cells are involved in cellular activities that include RNA binding, receptor binding and kinase activity (Figure 1B) [34-36]. In addition, the positively and negatively controlled mRNA targets were significantly over represented in biological terms describing roles in cell adhesion, cell proliferation and angiogenesis (Figure 1C and Table S3). Moreover, the identified mRNAs encoded proteins that were enriched in several biological pathways that play critical roles in cancer progression such as: kinase signaling, cytoskeleton regulation and apoptosis (Figure 1C and Table S3). Because several studies have identified the CELF1-transcriptome in multiple cell types, we sought to determine if CELF1 had a specific function in OSCC. We utilized the program ToppCluster [37] to identify shared as well as distinct CELF1 mediated biological processes amongst the various cell types. Compared to the CELF1 associated and controlled mRNA transcripts in T cells [31], HeLa cells [17], C2C12 cells [14], mouse muscle tissue and mouse cardiac tissue [38], CELF1 regulated mRNAs in OSCC are enriched in the biological processes related to mRNA translation and pathways involved in nonsense mediated decay (Table S4). Conversely, shared biological processes between the studies include, but are not limited to, regulation of cell death and cell cycle (Table S4). Although, this comparison is not ideal as previous CELF1 sequencing studies were mainly RIP-seq experiments, our analysis provides a preliminary assessment of a CELF1 specific function in OSCC.

To confirm our transcriptome analysis, we used the following criteria to select a subset of mRNAs for validation by qRT-PCR: (1) the mRNA transcripts exhibited a greater than 2-fold differential expression between control and CELF1 knockdown cells, (2) a putative GRE sequence was present in either the 5′ or 3′ UTR and (3) the mRNA transcripts encoded proteins that have significant biological role in cancer. Based on these criteria, we chose to measure the relative mRNA expression of eight up regulated, twelve down regulated and two neutral target mRNAs using transcript specific primers (Table S5). As an example, the RNA-seq read counts of *TIMP4* (up regulated), *IL24* (down regulated) and *PARP2* (unchanged) mRNAs are shown in Figure S1A. Our qRT-PCR analysis confirmed that 75% (15/20) of the mRNA targets chosen for validation were regulated by CELF1 (Figure 1D). Although 5 mRNA targets (*HDAC9*, *TGFBI, ID2*, *MXI1* and *PRSS21*) did not exhibit statistically significant changes in expression, due to experimental variation, the trend in their relative mRNA levels were similar to fold change values observed in our RNA-seq analysis. We have also measured mRNA expression of the 15 significant targets in UMSCC-11B and SCC-15 oral cancer cell lines treated with CELF1 siRNA, using qRT-PCR (Figure 1E). Seven out of 15 mRNAs in UMSCC-11B cells exhibited significant differential expression as a function of CELF1 protein levels. In addition, although not significant, three mRNA targets (*ANGPTL4*, CCL20 and *MAPK3*) displayed altered expression patterns similar to that observed in UMSCC-74B cells, as a function of CELF1 protein (Figure 1E). In SCC-15 cells five out of 15 mRNAs exhibited significant expression differences as a function of CELF1 protein levels. Also, three mRNAs *CXCL3, SERPINE2* and *TIMP4* although not significant displayed similar patterns of expression observed in UMSCC-74B cells. Thus, our RNA-seq data is an adequate representation of the CELF1 regulated transcriptome in oral cancer cells.

Our previous study determined that CELF1 protein and mRNA expression was elevated in HNSCC tumor samples compared to adjacent normal tissues [27]. Therefore, to establish if our panel of 15 CELF1 regulated mRNA targets in oral cancer cells were aberrantly expressed in human head and neck squamous cell carcinoma tumor tissue samples in comparison to normal specimens, we queried the TCGA (The Cancer Genome Atlas) HNSCC dataset using the UCSC cancer genomics browser [39]. *ESM1*, *IL8*, *CCL20*, *CXCL3*, *IL1RAP, IL24*, *CCNA1*, *SERPINE2* and *MMP1* mRNAs were down regulated in normal tissues in comparison to tumor samples (Figure 1F). Conversely, *KLF4*, *CXCL2, MAPK3* and *TIMP4* were up regulated in normal samples compared to tumor tissues (Figure 1F). In addition, CELF1 mRNA was elevated in tumor samples in comparison to normal tissues (Figure 1F). Collectively, these data suggest that the CELF1 regulated mRNAs may contribute to HNSCC development or progression. The remaining mRNA targets: *ANGPTL4* and *CXCL1* did not exhibit a dramatic alteration in mRNA expression (Figure 1F). Since, CELF1 mRNA and protein levels are known to be decreased in normal versus tumor tissues, the pattern of expression observed for *ESM1*, *KLF4*, *IL8*, *CCL20*, *IL24*, *CCNA1*, *TIMP4* and *MMP1* mRNAs, suggest that CELF1 may be a regulator of these targets in HNSCC. To investigate if CELF1 controls *ESM1*, *KLF4*, *IL8*, *CCL20*, *IL24*, *CCNA1*, *TIMP4* and *MMP1* mRNAs through a direct or indirect interaction we performed ribonucleoprotein immunoprecipitation (RNP-IP) using UMSCC-74B cells as our model cell line and quantified the associated mRNAs using qRT-PCR. *ESM1*, *IL8*, *IL24*, *CCNA1* and *TIMP4* were enriched 7-fold, 3-fold, 6-fold, 5-fold and 35-fold respectively, in CELF1 immunolysates (Figure 1G and Figure S1B). However, *CCL20*, *KLF4* and *MMP1* were not associated with CELF1 compared to IgG control (Figure 1G and Figure S1B). These data suggest that CELF1 regulates the expression of *IL8*, *IL24*, *CCNA1* and *TIMP4* directly, yet *CCL20*, *KLF4* and *MMP1* regulation may occur through the interaction of CELF1 and an intermediary protein such as another RNA binding protein or forms a ribonucleoprotein complex with other proteins.

### CELF1 regulates alternative splicing in oral cancer cells

**Figure 2:**
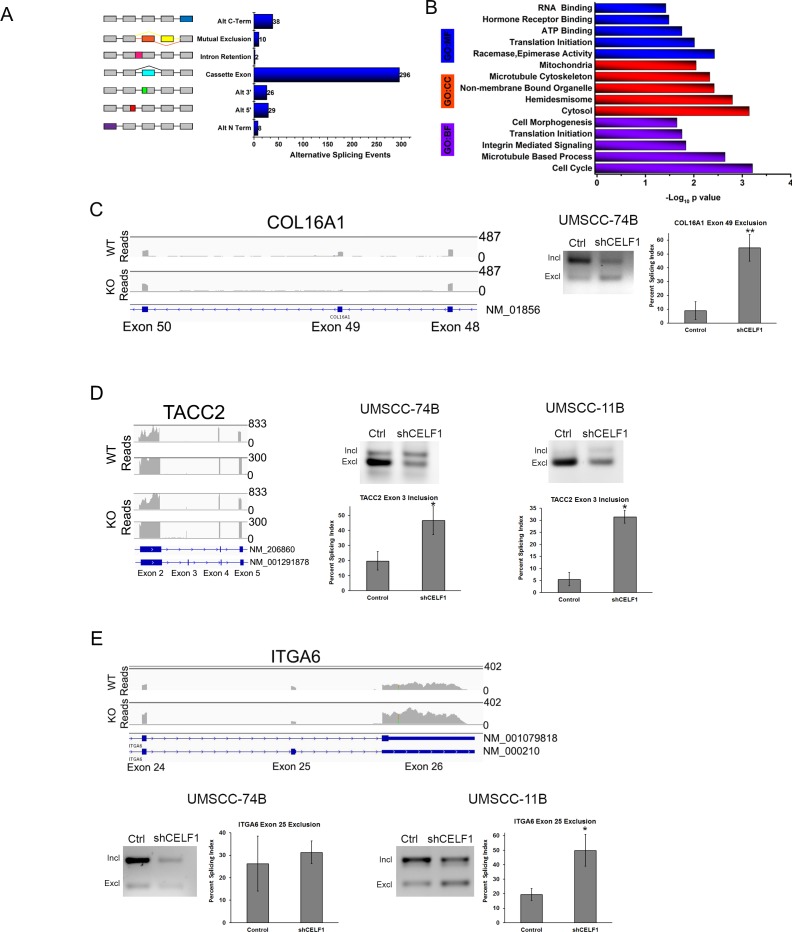
CELF1 protein expression affects alternative splicing **A.** Quantitation of CELF1 controlled alternative splicing events. **B.** Enrichment analysis of CELF1 regulated alternatively spliced genes. Illustration of alternative splicing of **C.** COL16A1 **D.** TACC2 **E.** ITGA6 using integrative genomics viewer. Numbers represent scale for transcript read counts. Scale for WT and KO reads are equivalent. Lower track represents zoomed in region for both TACC2 WT and KO reads. (Right) PCR agarose images of splicing events in UMSCC-74B and 11B oral cancer cells treated with control shRNA or CELF1 shRNA. Bar graph is quantitation of percent splicing index value. Data represented as mean ± SD; *N* = 3 **p value < 0.01; ***p value < 0.005.

The complexity of the human proteome is derived from the splicing of ∼90% of the estimated 21,000 coding genes present in the human genome [40]. Poorly regulated alternative splicing events can cause the development of disease [41, 42]. In DM1, phosphorylation of CELF1 by PKC increased CELF1 protein levels and subsequently promoted CELF1 mediated splicing events [43]. Although it was determined that the splicing of the *IR* (insulin receptor), *cTnT* (cardiac tropinin T), *CLC-1* (chloride channel type 1) and *PKM* (pyruvate kinase) by CELF1 can promote insulin resistance, cardiac abnormalities and muscle wasting in DM1 disease [22-25], the identity of the CELF1 cancer associated pre-mRNA splicing targets remain elusive. Therefore, to investigate the role of CELF1 in cancer associated alternative splicing, we used our RNA-seq transcriptome data to analyze alternative splice variants in sicontrol and siCELF1 transfected oral cancer cells. The AltAnalyze software uses two algorithms to quantify alternative splicing events, ASPIRE and splicing index. ASPIRE determines alternative splicing events by comparing the levels of the alternatively expressed exons and exon-exon junctions. The splicing index algorithm derives alternative splicing events by normalizing single exon expression to total gene expression levels [44]. The AltAnalyze algorithms identified 315 alternative splicing events, which corresponded to 282 genes (Table S6). The most prevalent alternative splicing event regulated by CELF1 was cassette exon skipping and inclusion (Figure 2A).

The top three significantly over represented biological functions of the 282 splicing targets were regulation of the cell cycle, microtubule based processes and translation initiation (Figure 2B and Table S7). Interestingly, the role of CELF1 in cell proliferation has not been previously studied in depth; therefore, we focused on the CELF1 mediated alternative splicing events of three pre-mRNAs known to play a role in cell growth (*COL16A1*, *TACC2* and *ITGA6*). Most notably, compared to the expression of flanking exons in UMSCC-74B cells, CELF1 depletion caused a 5-fold increase in the exclusion of exon 49 in *COL16A1* (collagen type XVI), which encodes an extracellular matrix protein involved in oral cancer cell proliferation and glioma cell invasion (Figure 2C) [45, 46]. Unfortunately, we were unable to detect a PCR product for *COL16A1* in UMSCC-11B cells. The loss of exon 49 in *COL16A1* according to our analysis, results in increased expression of a *COL16A1* noncoding RNA product with the Ensembl transcript ID ENST00000488128 and a loss of expression of the APPRIS predicted principle isoform ENST00000373672 (NM_001856) in CELF1 knockdown cells. TACC2 is a member of the transforming acidic coiled coil family of proteins known to associate with centromeric microtubules and promote cell cycle progression [47]. In CELF1 reduced cells, inclusion of exon 3 in *TACC2* transcript, ENST00000369004, was increased approximately 2.0-fold and 6.0-fold in UMSCC-74B and UMSCC-11B cells, respectively, compared to control cells (Figure 2D). Exon 3 does not encode for any known protein domains; therefore, the effect of this *TACC2* splicing event on TACC2 protein and function is unknown. *ITGA6* (integrin alpha 6) is known to exist as two variants in mammalian cells, α6A and α6B [48]. The difference in these two variants is the expression of exon 25, which encodes a cytoplasmic integrin domain. According to our RNA-seq data, the loss of CELF1 promoted the exclusion of exon 25 in *ITGA6* (Figure 2E) a regulator of cell motility, invasion and proliferation [49, 50]. Exclusion of exon 25 due to the loss of CELF1 protein could result in an ITGA6 protein with reduced signaling capacity in OSCC cells [50]. In UMSCC-11B cells treated with CELF1 shRNA, we measured ∼2.5 fold increase in the exclusion of exon 25 compared to control cells. However, we did not detect a significant difference in the formation of the *ITGA6* α6B variant (exon 25 excluded), by PCR in UMSCC-74B cells, in comparison to control. Lastly, we used the TCGA SpliceSeq database provided by MD Anderson Cancer Center [51], to determine if these splicing events were present in human HNSCC tumors. Our preliminary analysis suggests that there is a difference in expression, although modest, for the *COL16A1*, *TACC2*, and *ITGA6* CELF1-mediated splicing events in HNSCC tumors compared to normal samples (Figure S2).

### Suppression of CELF1 reduces tumor volume *in vivo*

**Figure 3:**
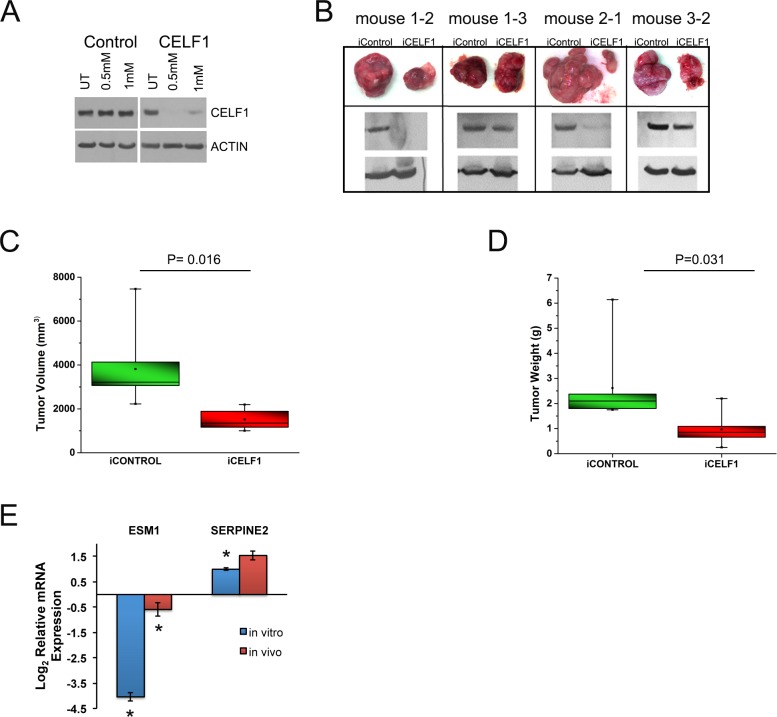
Reduction of CELF1 decreases tumor volume *in vivo* **A.** Western blot analysis of CELF1 protein expression day 6 post treatment of UMSCC-74B cells with 0.5mM and 1mM IPTG. **B.** Representative tumor images of four nude mice treated with 21mM IPTG/5% glucose in drinking water. *N* = 7 mice **C.** Quantitation of iCELF1 and iControl UMSCC-74B cell tumor volumes. *N* = 7; median (horizontal line); box (interquartile range); whiskers (minimum and maximum) mean (point inside box). **D.** Measurement of tumor weight. *N* = 7; median (horizontal line); box (interquartile range); whiskers (minimum and maximum) mean (point inside box). **E.** Quantitative real time PCR analysis of ESM1 and SERPINE2 mRNA levels in UMSCC-74B cells in culture and in xenograft tumors. Bars represent mean ± SE **p value < 0.05.

To determine if CELF1 protein expression affected tumor growth *in vivo*, we created UMSCC-74B cells stably transduced with inducible control or CELF1 shRNA vectors. In cultured cells, maximal reduction of CELF1 levels occurred at 6 days post treatment with 0.5mM IPTG (Figure 3A). To determine the role CELF1 protein expression plays in tumor growth, we injected inducible control shRNA (right-side) and CELF1 shRNA (left-side) UMSCC-74B clones into the flanks of nude mice (Figure 3B). Treatment of mice with 21mM IPTG in the drinking water led to a reduction in tumor volume for those cells stably transduced with inducible CELF1 shRNA in comparison to control cells, 1636mm^3^ and 3473mm^3^, respectively (Figure 3C). In addition the average weight of the CELF1 shRNA tumors was significantly less in comparison to control cells, 0.96g and 2.61g, respectively (Figure 3D). To determine if the expression of our CELF1 regulated panel of mRNAs was altered *in vivo*, we isolated RNA from both control and CELF1 reduced xenograft tumors and analyzed mRNA expression using qRT-PCR. We found that *ESM1,* was significantly reduced both *in vitro* and *in vivo* as a function of CELF1 protein levels (Figure 3E). Although, *SERPINE2* had a similar expression pattern both *in vitro* and *in vivo*, the *in vivo* expression was not significant when compared to control tumors (Figure 3E). In addition, we compared proliferation and angiogenesis markers between shcontrol and shCELF1 tumors and found no difference in the number of Ki67 and CD31 positive cells, respectively (Figure S3). The reduction in tumor burden displayed by the CELF1 shRNA inducible clones suggest that CELF1 plays a major role in tumor growth possibly through controlling several genes and pathways described in Figure 1C and promoting cell death through caspase-3, which was previously described by our laboratory in UMSCC-74B cells [27].

### Overexpression of CELF1 in immortalized human oral keratinocytes enhances cell survival

**Figure 4:**
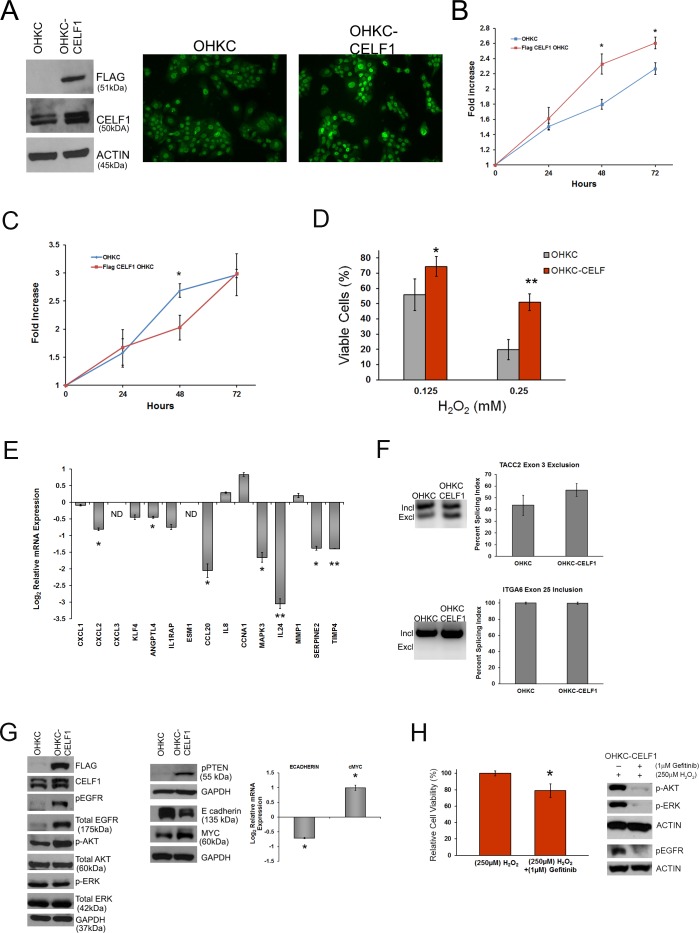
CELF1 overexpression in immortal human oral keratinocytes protects against oxidative stress **A.** (Left) Western blot analysis of Flag-CELF1 overexpression in OKF6-tert (OHKC) cells (Right) Immunocytochemistry of CELF1 expression in control OHKC cells and CELF1 overexpressing OHKC cells (OHKC-CELF1). **B.** Measurement of control OHKC and OHKC-CELF1 viability measured by MTT. Data represented as mean ± SD. *p value < 0.05; *N* = 3 **C.** Measurement of control OHKC and OHKC-CELF1 cells using CyQuant assay. Data represented as mean ± SD. **p value < 0.05; *N* = 3 **D.** Treatment of OHKC and OHKC-CELF1 cells for 24 hours with H2O2. Data represented as mean ± SD. *p value < 0.05; ***p value < 0.005; *N* = 3 **E.** Expression analysis of 15 CELF1 mRNA targets in control OHKC and OHKC-CELF1 cells using qRT-PCR. ND: Not detected in control OHKC Bars represent mean ± SE. **p value < 0.05; ***p value < 0.005; *N* = 3 **F.** Measurement of OHKC and OHKC-CELF1 splicing of TACC2 and ITGA6 using semi-quantitative PCR. Data represented as mean ± SD; *N* = 3. **G.** Western blot analysis of cell proliferation associated proteins in OHKC and OHKC-CELF1 cells. **H.** (Left) Relative OHKC-CELF1 cell survival post 24 hours treatment with H2O2 and Gefitinib. (Right) Western blot analysis of downstream EGFR molecules affected by Gefitinib treatment. Data represented as mean ± SD. **p value < 0.05; *N* = 3.

To support our findings that CELF1 controls gene expression, cell growth and survival, we overexpressed a FLAG- tagged CELF1 construct in OKF6-TERT1 human oral keratinocytes (OHKC) (Figure 4A). First, to determine if exogenous expression of CELF1 altered cellular function, we measured cell viability in the OHKC-CELF1 cells using MTT. We detected a significant increase in the viability of OHKC-CELF1 cells at 48 and 72 hours post cell seeding (Figure 4B) compared to control OHKC, suggesting that CELF1 enhances cell growth. To determine if CELF1 controlled cell proliferation, we utilized the CyQuant proliferation assay to measure the number of cells over a three day observation period (Figure 4C). Overall, we did not detect a significant difference in the number of OHKC-CELF1 cells compared to control OHKC-cells (Figure 4C). The increased cell viability measured by MTT would suggest that the OHKC-CELF1 cells have altered cellular metabolic activity. Changes in cell metabolism are known to alter cell survival. CELF1 is known to regulate cancer cell apoptosis [17, 26, 27, 30] hence, we sought to determine if CELF1 expression would protect OHKC-CELF1 cells against environmental stress induced cell death. OHKC and OHKC-CELF1 cells were treated with 0.125mM and 0.250mM H_2_O_2_ (hydrogen peroxide) for 24 hours to mimic oxidative damage. OHKC-CELF1 cells exhibited a statistically significant increase in protection against oxidative damage when compared to control OHKC cells (Figure 4D). We evaluated our panel of 15 mRNAs and three alternative splicing targets in OHKC and OHKC-CELF1 cells using qRT-PCR and semi-quantitative PCR, respectively. We hypothesize, that if these CELF1 controlled mRNAs were important for tumor formation and/or progression, OHKC-CELF1 cells would have distinct relative mRNA and splice variant expression levels in comparison to control OHKC cells. The expression levels of seven out of 15 mRNAs targets were significantly altered in OHKC-CELF1 cells compared to OHKC control cells (Figure 4E). We measured the formation of the alternative exons for *TACC2* and *ITGA6* in OHKC and OHKC-CELF1 cells; we were unable to detect *COL16A1* splice variants in control OHKC cells. Based on our analysis in OSCC cells, over expression of CELF1 in OHKC cells should promote the exclusion of exon 3 in *TACC2*. Although, an enhancement in the exclusion of exon 3 in *TACC2* was observed in OHKC-CELF1 cells compared to control OHKC cells, this difference was not statistically significant (Figure 4F). The exclusion of exon 3 in *TACC2* may require additional features such as interaction with other RBPs, formation of mRNP complex or cancer specific signaling cascades. In addition, we did not detect the *ITGA6* α6B variant in both control and OHKC-CELF1 cells. Therefore, we could not measure a change in the inclusion of *ITGA6* exon 25 as a function of CELF1 expression (Figure 4E). Altogether, these experiments support CELF1 playing a role in cell survival through regulation of gene expression.

Finally, to determine how CELF1 is regulating cell survival, we analyzed the expression of key intracellular signaling molecules known to control cell survival. Interestingly, overexpression of CELF1 led to an increase in total EGFR as well as phosphorylated (Y1148) EGFR protein (Figure 4G). The elevated EGFR levels resulted in enhanced expression of downstream EGFR targets such as pAKT (S473) and pPTEN (S380) (Figure 4G). Surprisingly, phosphorylated ERK levels were unchanged between OHKC and OHKC-CELF1 cells, suggesting that EGFR signaling may promote the activation of other MAPK such as p38 and JNK (Figure 4G). In addition, we measured cMYC expression in the OHKC-CELF1 cells. The cMYC oncogene is known to regulate cell proliferation in a variety of cell types and can also potentiate the survival response of cells exposed to oxidative stress [52]. Overexpression of CELF1 increased cMYC protein and mRNA levels in OHKC-CELF1 cells compared to control (Figure 4G). Lastly, E-cadherin, is known to regulate cell proliferation and is a key player in the epithelial-mesenchymal transition (EMT); however, the loss of E-cadherin can protect cells from the induction of apoptosis [53-55]. Therefore, we measured E-cadherin protein and mRNA levels in OHKC and OHKC-CELF1 cells. OHKC-CELF1 cells had a marked reduction in E-cadherin protein and ∼1.5 fold reduction in E-cadherin mRNA compared to OHKC control cells (Figure 4G). Interestingly, it was discovered that E-cadherin can regulate EGFR in HNSCC cell lines [53] and that CELF1 can regulate E- cadherin protein levels in lung squamous cell carcinoma cells [30]; thus, CELF1 may control cell survival through coordinated regulation of EGFR, E-cadherin and cMYC expression in OHKC-CELF1 cells. To investigate if EGFR signaling promoted CELF1 induced cell survival we treated OHKC-CELF1 cells with 0.250mM H_2_O_2_, in the presence or absence of the EGFR small molecule inhibitor Gefitinib (Figure 4H). In the presence of 1μM Gefitinib, OHKC-CELF1 cells treated with H_2_O_2_ exhibited a significant ∼20% reduction in cell survival compared to OHKC-CELF1 cells treated with DMSO and H_2_O_2_ (Figure 4H). These observations suggest that overexpression of CELF1 in OHKC cells protects cells from apoptosis through modulation of EGFR signaling.

## DISCUSSION

Although there have been treatment advances for HNSCC, the overall survival rate has not increased dramatically over the last 30 years; therefore discovery of new molecular determinants of HNSCC, may be useful for the development of more effective therapeutic agents. Intriguingly, CELF1 is a broadly expressed member of the CUGBP ELAV-like family of RBPs and is overexpressed in multiple cancers including lung and oral cancer [26, 27, 30]. Our previous report indicated that CELF1 accumulation correlated with cancer stage, suggesting that CELF1 may play an important role in oral cancer progression [27]. In this study, utilizing next generation sequencing in CELF1 depleted oral cancer cells, we identified mRNA targets that were both directly and indirectly controlled by CELF1. Therefore, we were able to comprehensively assess CELF1-mediated gene expression patterns in oral cancer cells.

In this report, we demonstrated that in oral cancer cells CELF1 controls the expression of 1283 mRNAs that were enriched in biological terms associated with cell proliferation and apoptosis, which was not surprising. However, CELF1 regulated mRNAs were also enriched in cellular processes such as angiogenesis and cell signaling, which are important for tumorigenesis and/or progression. Thus, CELF1 is a critical regulator of tumor cell biological processes. Querying the TCGA HNSCC dataset we identified 8 potential mRNA targets (*ESM1*, *KLF4*, *IL8*, *CCL20*, *IL24*, *CCNA1*, *TIMP4* and *MMP1*) from our validated 20 mRNA panel, which were aberrantly expressed in HNSCC and controlled by CELF1 in oral cancer cells. *MMP1*, *IL24* and *CCL20* are mRNAs that are well studied for their role in cancer; however the remaining mRNAs have not been characterized for their potential role in HNSCC tumorigenesis and/or tumor progression and warrant further study. Interestingly, *IL24/mda-7* is classified as a tumor suppressor in several cancers including: melanoma, gall bladder and lung [56-59]. *IL24* mRNA and protein levels are low in cell lines derived from these tumors and adenoviral overexpression of IL24 increased tumor cell apoptosis and decreased tumor cell migration and invasion [57-59]. Paradoxically, *IL24* mRNA is up regulated in HNSCC tumor samples in comparison to normal tissues according to the 541 patient TCGA HNSCC dataset, this suggests that *IL24* may promote HNSCC tumorigenesis and/or tumor progression. A recent publication identified *IL24* as a potential biomarker for advanced disease in HNSCC; however, the exact mechanism of *IL24* mRNA regulation in oral cancer cells is currently unknown. Therefore, future studies from our laboratory are focused on defining the mechanism of CELF1 regulation of *IL24* mRNA expression in HNSCC.

The expression of CELF1 in the nuclear and cytoplasmic subcellular compartments orchestrates two different cellular mRNA processes. Nuclear CELF1 is well characterized for its role in alternative pre-mRNA splicing in a variety of model systems [60-63]; however, its role in pre-mRNA splicing in cancer is understudied. The major CELF1-mediated pre-mRNA splicing event is cassette exon exclusion/inclusion; this observation is consistent with HITS-CLIP splicing analysis of CELF1 in C2C12, muscle cells [32]. Interestingly, the loss of CELF1 protein promotes the alternative splicing of *COL16A1*, resulting in the expression of a noncoding *COL16A1* RNA product. It is well established that RBPs, in conjunction with noncoding RNAs such as long noncoding RNAs, are capable of regulating several steps within the post-transcriptional gene regulatory process. For example, pseudogenes or long intergenic noncoding RNAs (lincRNAs) can exert their control of gene expression through acting as miRNA sponges [64, 65]. Therefore, the formation of the *COL16A1* noncoding RNA by CELF1 mediated alternative splicing events, highlights a novel mechanism by which CELF1 may control gene expression.

Suppression of CELF1 protein *in vivo* reduced tumor growth clearly demonstrating CELF1's role in tumor progression. These findings support that the CELF1 mediated gene expression program acts as a promoter of pro-tumorigenic genes in tumors of oral epithelial origin. Hence, a potent pharmacological intervention disrupting the CELF1-mRNA interfaces could abrogate tumor cell growth. CELF1 is widely studied for its role in normal muscle development [24, 25, 60, 66]. Mice overexpressing CELF1, display phenotypic features similar to human myotonic dystrophy disease [67]. CELF1 protein levels are often elevated in human pathologies, our data revealed that enhanced CELF1 levels in the immortalized keratinocytes protected cells against oxidative stress-induced cell death. These observations support CELF1 as a regulator of cell viability and are in accordance with previous reports where reduction of CELF1 protein in cancer cells induced apoptosis [8, 30]. Intriguingly, we have observed that overexpression of CELF1 altered EGFR receptor expression, causing activation of downstream targets of EGFR signaling. Previously, it was determined that EGFR is capable of activating CELF1 [68] in contrast, our observation is the first to show that CELF1 can regulate EGFR expression, supporting CELF1 as a pro-survival protein. Elevated expression of CELF1 also caused a marked decrease in the expression of E-cadherin, suggesting that CELF1 expression may influence epithelial-mesenchymal transition (EMT). Recently, CELF1 was shown to affect E-cadherin levels in A549 lung cancer cells, however, the mechanism of CELF1 regulation of E-cadherin remains to be elucidated [30]. In addition, CELF1 was able to control the oncogene MYC expression in the immortalized keratinocytes. A CELF1-MYC interaction was established in gut epithelial cells where it was observed that CELF1 can regulate proliferation through competing with the HuR RNA binding protein to regulate MYC protein translation [69]. In that study, CELF1 was a negative regulator of MYC protein expression, however in our system CELF1 promotes MYC protein levels possibly through stabilizing *MYC* mRNA. Although, the CELF1 regulatory effect on *MYC* mRNA may be cell type dependent, it is evident that the CELF1-*MYC* interaction plays an important role in cell viability. Thus, further investigation of how CELF1 regulates cell survival and possible EMT through control of MYC and E-cadherin, warrants further investigation.

## MATERIALS AND METHODS

### Cell lines, qRT-PCR and primers

UMSCC-74B (tongue squamous cell carcinoma), UMSCC-11B and SCC-15 were maintained in DMEM supplemented with 10% FBS and 2% penicillin/streptomycin. OKF6-TERT1 human oral keratinocytes were maintained in keratinocyte serum free medium supplemented with 1ng/ml EGF, 30μg/ml bovine pituitary extract and 0.9mM CaCl_2_ (Life Technologies). All qRT-PCR was performed using Applied Biosystem StepOne Plus system with RT^2^ SYBR green ROX PCR Mastermix (SA Biosciences). Transcript specific primers were purchased from Integrated DNA Technologies utilizing the PrimeTime qPCR Assay Tool. GAPDH primer was purchased from Sigma. Primer sequences and assay identification numbers are available in Supplemental Experimental Procedures.

### Western blot analysis

Cells were lysed using RIPA buffer and proteins were separated using SDS-PAGE. Proteins were transferred to PVDF membrane and incubated with primary antibodies at 4°C overnight. Membranes were washed three times with TBST and incubated with secondary antibody for 1 hour at room temperature. Proteins were visualized using enhanced chemiluminescence. All primary antibodies were used at a 1:1000 dilution, except GAPDH and actin were used at 1:5000 dilution. Actin (Sigma Aldrich); CELF1 (Millipore) and remaining antibodies were purchased from Cell Signaling Technologies. Secondary antibodies were used at 1:5000 dilutions and purchased from GE Healthcare.

### Next generation sequencing, alternative splicing and bioinformatic analyses

RNA was isolated form sicontrol or siCELF1 treated UM74B and subjected to RNA quality assessment using Agilent Bioanalyzer. RNA-seq libraries compatible with Illumina PET sequencing were constructed for each condition. Each library was run on a single 100 base indexed PET flow cell lane. Sequencer FASTQ files were quality checked and filtered using a standard Illumina pipeline and a proprietary Genotypic SeqQC V2.1 tool. Reads were aligned individually to human whole genome (Hg19 build) using TopHat v0.1.0. Transcript identification and relative abundances were determined using cufflinks v1.1.0. Novel isoform prediction and isoform comparison was performed using cuffcompare v1.1.0 and differential gene expression was quantified using cuffdiff v1.1.0. (See supplemental materials for detailed pipeline of analysis). Gene enrichment analysis and network visualization were performed using the ClueGo and CluePedia plugins for Cytoscape. Domains of spliced transcripts were identified using Pfam 27.0 (HHMI). ToppCluster with default parameters was used for multiple gene list biological enrichment comparison. The 3′UTRs for the 1174 genes were extracted using ensembl biomart. A perl string match method in scalar mode was used to count the instances of the CELF1 sequence patterns in each of 3′ UTRs.

### Semi-quantitative PCR

CELF1 alternative spicing targets were amplified with designed exon specific primers using a standard thermocycler PCR program. Percent splicing index was calculated by dividing the included or excluded exon band intensity by the sum of both included and excluded exon band intensities. Quantitation of PCR products was performed using Image J software.

### siRNA transfection and shRNA transduction

UM74B cells were transfected with 20nM CELF1 siRNA (Dharmacon) or 20nM control siRNA (Qiagen) using HiPerfect transfection reagent (Qiagen) following the manufacturer's protocol. CELF1 shRNA and control shRNA plasmids were purchased from Sigma. Cells were transduced with lentiviral particles at a MOI of 50 in medium supplemented with 8μg/ml polybrene.

### Ribonucleoprotein immunoprecipitation (RNP-IP)

RNP-IP experiments were performed as described previously [14, 27]. Briefly, UM74B cells grown under normal conditions were UV cross-linked (200mJ/cm^2^) and lysed with polysome lysis buffer. A minimum of 1mg of total protein was added to Protein A/G (Santa Cruz Biotechnology) beads coated with 1.4μg of CELF1 (EMD Millipore) or isotype control antibody (Santa Cruz Biotechnology). Lysates were incubated with antibody for 2 hours at 4°C and RNA was isolated using Trizol Reagent (Life technologies).

### Inducible shRNA CELF1 xenograft mouse studies

Six week old male nude mice (Charles River Laboratories) were injected subcutaneously in the right flank with 5×10^6^ UMSCC-74B cells stably transduced with control 3XlacO inducible shRNA and left flank of the same mouse with CELF1 3XlacO inducible shRNA (Sigma Aldrich) resuspended in 20% Matrigel. One-day post injection mice were treated with 21mM IPTG (Lab Scientific) and 5% glucose in the drinking water. Tumor volumes were measured twice weekly using digital calipers and mice were sacrificed when tumor volumes exceeded 3000mm^3^. Tumor volume formula = 0.5*(L*W^2^) W = shortest side L = longest side. (See supplemental materials and methods for statistical analysis).

### Cell viability assay and H_2_O_2_ treatment

Control or CELF1 overexpressing OKF6-TERT1 cells were plated in 96-well plates. For viability assay cells were treated with MTT and absorbance was read at 570nm every 24 hours for 3 days. Cyquant assay was performed similarly to MTT assay with exception of cyquant reagent was added to cells for 2hours and fluorescence was measured at Ex. 485/Em. 515. For oxidative stress cells were plated in 96-well plates and treated for 24 hours with 0.125mM and 0.250mM H_2_O_2_. After 24 hours MTT was added to the cells, the cells were lysed after 2 hours and absorbance readings at 570nm were taken. For treatment experiments cells were exposed to 1μM of Gefitinib in the presence of 0.250mM H_2_O_2_ for 24 hours.

### Statistical analysis

Quantitative real time PCR data were expressed as the log_2_ mean ± the standard error of the mean. Two-sample t-tests with unequal variances were used to assess differences between means. Results with p values less than 0.05 were considered significant. Realtime PCR analysis of mRNA target expression in xenograft tissues were expressed as log_2_ mean ± the standard error of the mean. For *in vivo* studies, analysis of paired tumor volumes (or tumor weights) comparing control to CELF1-injected flanks were performed using Wilcoxon signed-rank test. In addition, analysis of differences in gene expression comparing control to CELF1-injected flanks was performed using paired Wilcoxon signed-rank test. Results with p values less than 0.05 were considered significant. UCSC Cancer Genomics Browser statistical analysis of mRNA target expression in normal versus tumor samples was performed by creating two subgroups (solid tissue normal and primary tumor) and selecting Wilcoxon Test.

### Accession numbers

Transcriptome fast q files have been deposited into Gene Expression Omnibus (GEO) and can be accessed using the GEO number GSE57254.

## SUPPLEMENTARY MATERIAL FIGURES AND TABLES
















