# Supplementary material for: RNA-binding protein CELF1 promotes tumor growth and alters gene expression in oral squamous cell carcinoma
Source: Oncotarget. 2015 Oct 20;6(41):43620–34. doi: 10.18632/oncotarget.6204 (PMC4791255; doi:10.18632/oncotarget.6204)
Supplement: Supplementary file 1 [file oncotarget-06-43620-s001.pdf]

RNA-binding protein CELF1 promotes tumor growth and alters gene expression in oral squamous cell carcinoma

Supplementary Material

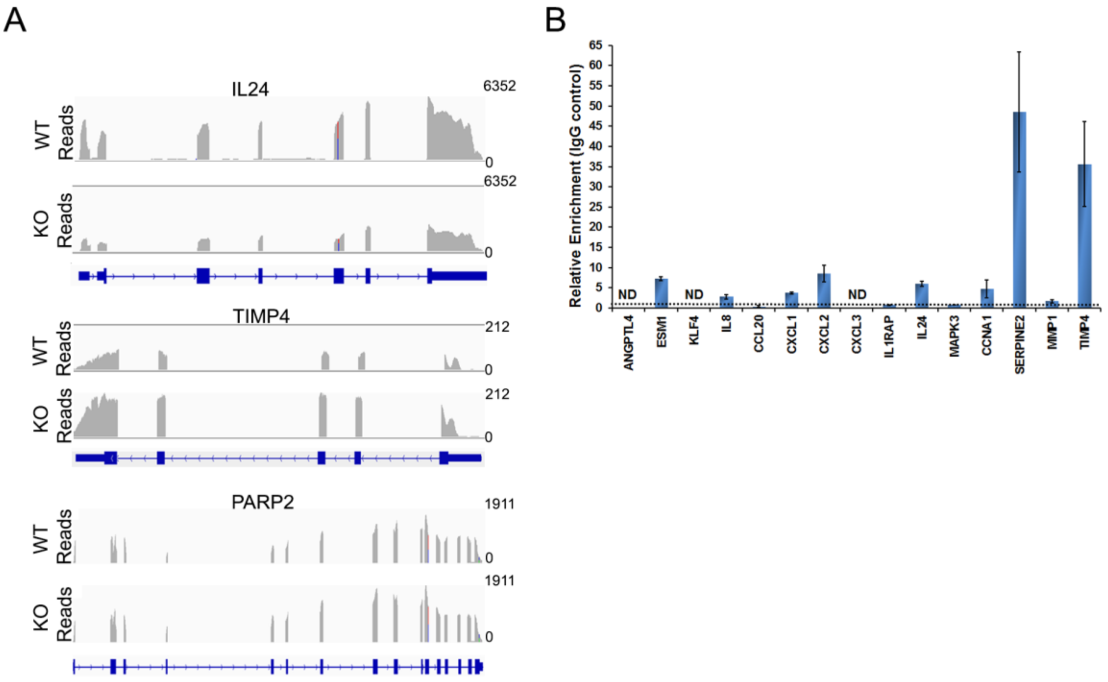

Supplemental Figure 1: (A) IGV (Integrated Genome Viewer) illustration of transcripts used in qRT-PCR validation. *IL24* down regulated, *TIMP4* upregulated and *PARP2* neutral. Numbers represent scale for transcript read counts. Scale for WT and KO reads are equivalent. (B) CELF1 ribonucleoprotein immunoprecipitation in UMSCC 74B cells. The association of the 15 panel mRNAs was measured using qRT-PCR.

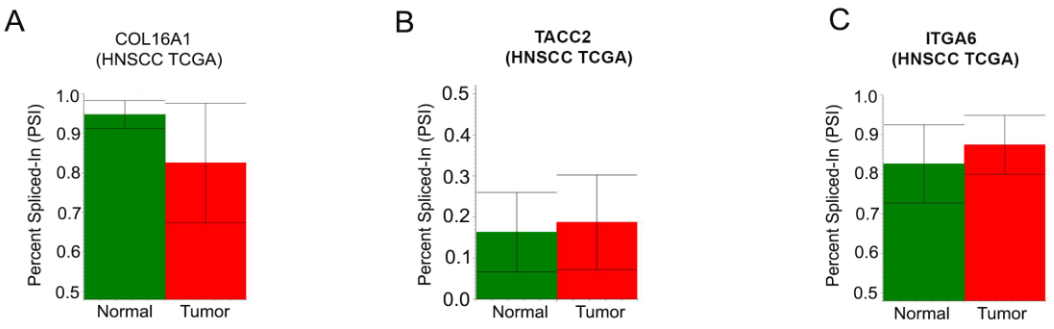

Supplemental Figure 2: HNSCC TCGA splicing analysis of (A) *COL16A1* (B) *TACC2* and (C) *ITGA6.* Data represented as mean ± SD.

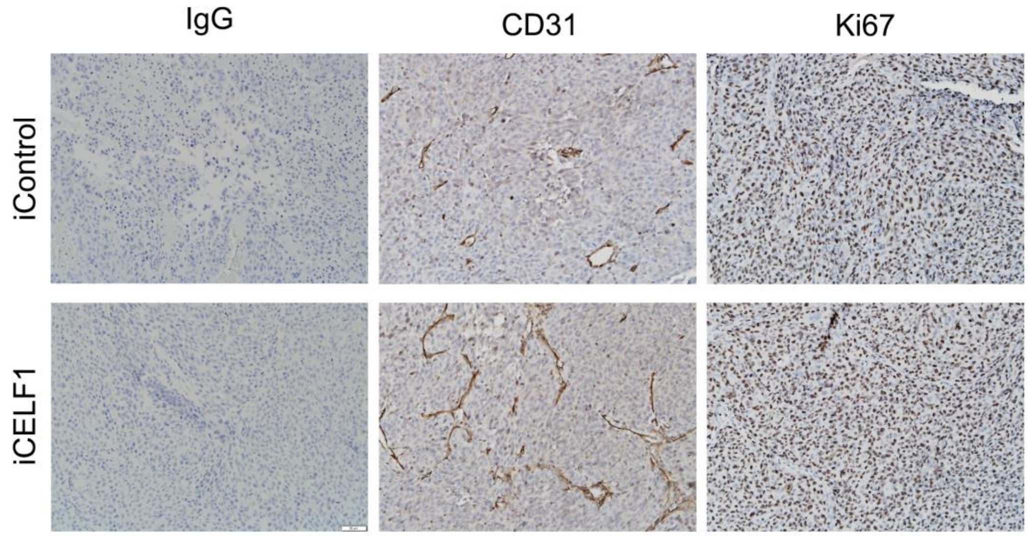

Supplemental Figure 3: Representative Immunohistochemical staining of Ki67 (proliferation marker), CD31 (endothelial cell marker) and IgG control of icontrol or iCELF1 tumors. 20X magnification. Scale bar 50µm.
